# Supplementary material for: Discontinuous epidemic transition due to limited testing
Source: Nat Commun. 2021 May 10;12:2586. doi: 10.1038/s41467-021-22725-9 (PMC8110767; doi:10.1038/s41467-021-22725-9)
Supplement: Supplementary file 1 — Supplementary Information [file 41467_2021_22725_MOESM1_ESM.pdf]

Discontinuous epidemic transition due to limited testing

Supplementary Information

Davide Scarselli[1]†, Nazmi Burak Budanur[1]†, Marc Timme[2], Björn Hof[1]*

[1] Institute of Science and Technology Austria, Am Campus 1, 3400 Klosterneuburg, Austria

[2] Chair for Network Dynamics, Center for Advancing Electronics Dresden (cfaed), Institute for Theoretical Physics and Center of Excellence Physics of Life, Technical University of Dresden, 01062 Dresden, Germany

*Correspondence to: bhof@ist.ac.at

†These authors contributed equally to this work

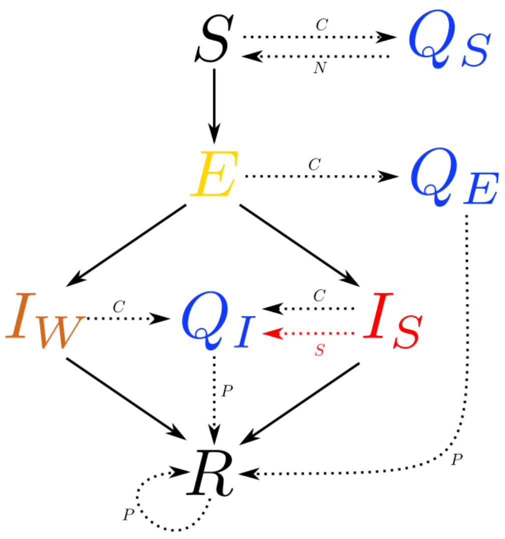

Supplementary Fig. 1 Transitions between possible states. Solid arrows denote the typical disease progression for an undetected individual, while dotted arrows denote possible events in a testing and quarantining scenario. In particular, S, E, I_W and I_S are quarantined if they are in contact with a known positive (arrow C), while I_S can be immediately identified and quarantined (arrow S). After testing negative Q_S reverts to S (arrow N) while Q_E, Q_I and R test positive and transform into R.

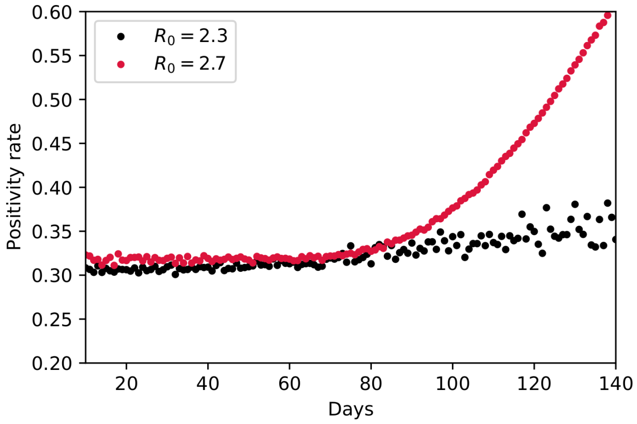

Supplementary Fig.2 Positivity rate under limited testing. Time evolution of the positivity rate (here defined as the ratio between new positive cases and the number of daily tests performed) for R_0=2.3 (black dots) and R_0=2.7 (red dots) with N_T=1000 daily available tests. The values of the positivity rate have been averaged over more than 100 simulations. The population is P=3162×3162≈10^7 people and epidemics start with 100 initial weak-symptom infectious.

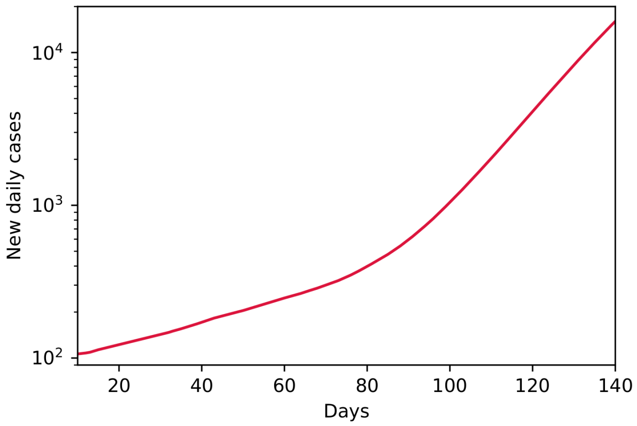

Supplementary Fig. 3 Faster than exponential growth. Daily new cases computed for R_0=2.7. Testing and quarantining are carried out at the same time with a capacity limit of N_T=1000 tested individuals per day. Initially the growth rate is constant and corresponds to an effective reproduction number R_t≈1.1 (cf. Fig. 2d). When the number of suspects reaches the test capacity (around day 70), Δ_{Test}=0 and the outbreak accelerates super-exponentially as the effective reproduction number increases up to R_t≈1.4 (cf. Fig. 2d). The values of daily new cases have been averaged over 100 simulations. The population is P=3162×3162≈10^7 and epidemics start with 100 initial weak-symptom infectious.

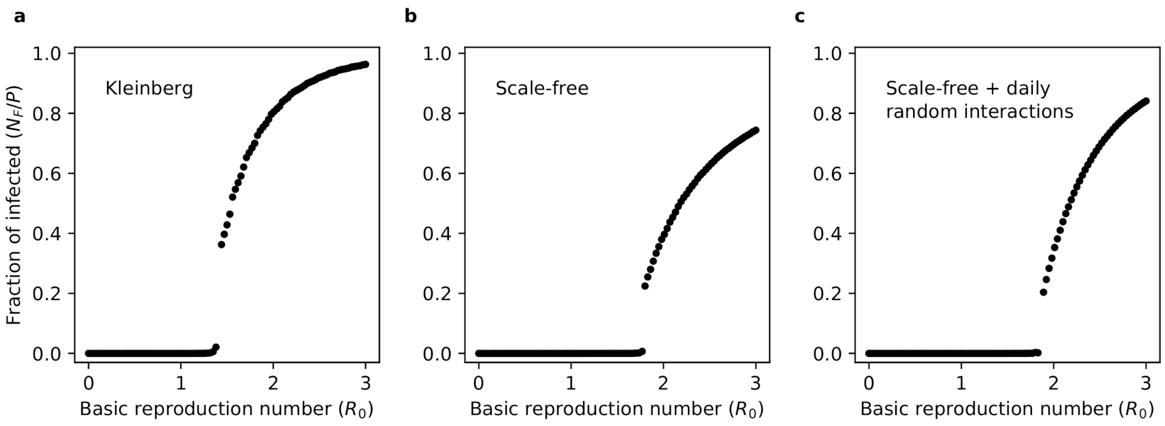

Supplementary Fig. 4 Network robustness. a Discontinuous epidemic transition for Kleinberg's Small World network. For this case the population is P=3162×3162 and epidemics start with 100 weak-symptom infectious. The ratio of weak-symptom cases is set to 50%. Strong-symptom cases are recognized with probability 0.5 and neighbors are traced with probability 0.5. The number of daily tested individuals is N_T=1000. **b** Discontinuous epidemic transition for the scale-free network. For this case the population is P=3162×3162 and epidemics start with 100 weak-symptom infectious. The ratio of weak-symptom cases is set to 50%. Strong-symptom cases are recognized with probability 0.5 and neighbors are traced with probability 0.8. The number of daily tested individuals is N_T=1000. **c** Discontinuous epidemic transition for the scale-free network with daily random interactions. For this case the population is P=3162×3162 and epidemics start with 100 weak-symptom infectious. The ratio of weak-symptom cases is set to 50%. Strong-symptom cases are recognized with probability 0.5 and neighbors are traced with probability 0.8. Random daily interactions are untraced. The number of daily tested individuals is N_T=1000.

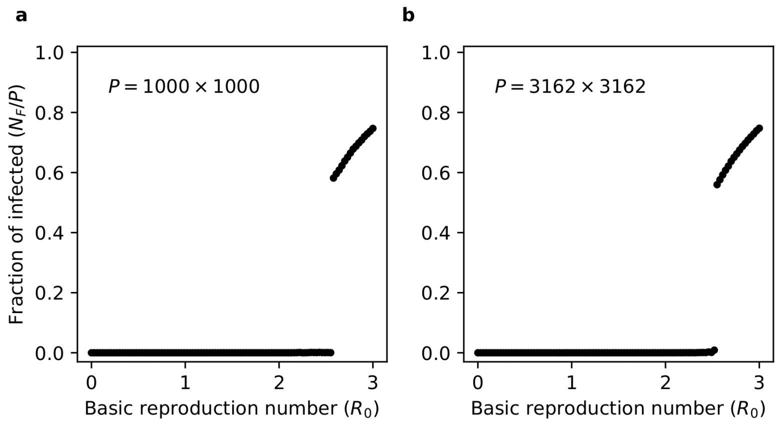

Supplementary Fig. 5 Effect of the domain size. a Discontinuous epidemic transition for a population P=1000×1000=10^6. The number of initially weak-symptom infectious is 10 and N_T=100. **b** Discontinuous epidemic transition for a population P=3162×3162. The number of initially weak-symptom infectious is 100 and N_T=1000. In both cases the ratio of weak-symptom cases is set to 50%. The discontinuous nature of the epidemic transition remains unaltered when the population size, test capacity and initial number of infectious are scaled by the same factor.

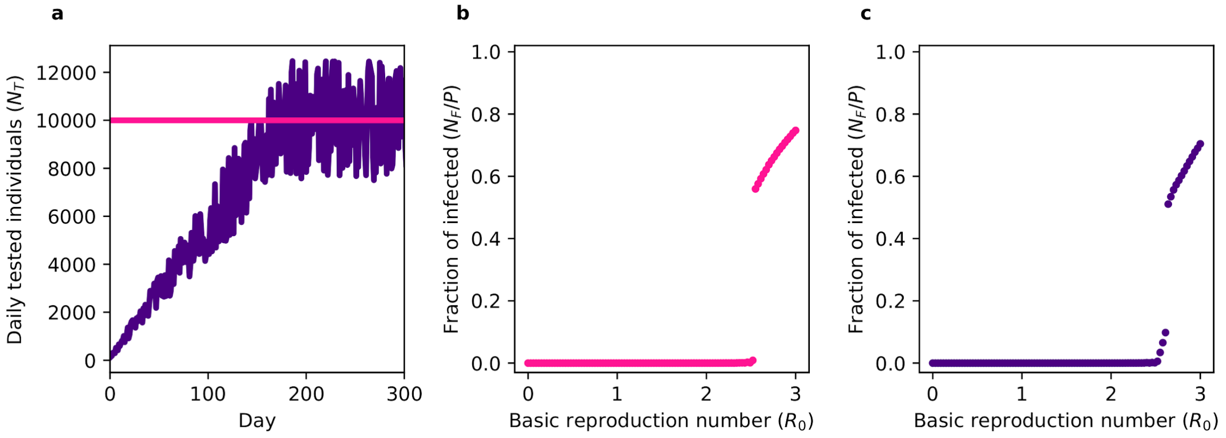

Supplementary Fig. 6 Effect of different testing scenarios. a Two testing scenarios are considered to show the robustness of the discontinuity with respect to different testing strategies. We assume a constant number of tests N_T=10000 (pink) and a linear increase from N_T=100 to N_T=10000 over 180 days, with in addition random fluctuations of 20% of the daily value (dark violet).

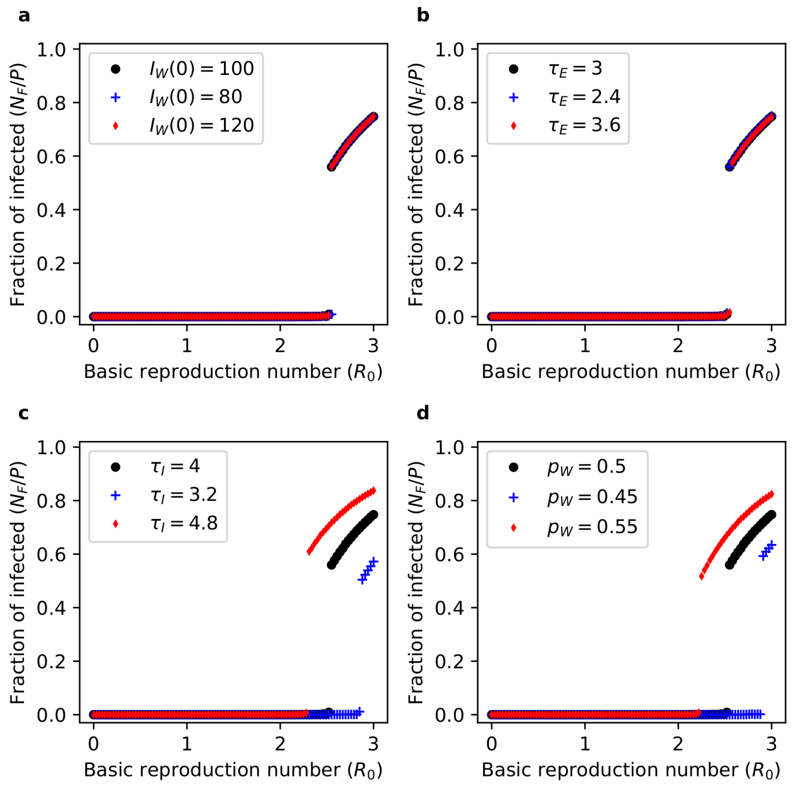

Supplementary Fig. 7 Robustness of the discontinuous transition against parameter perturbations. Shown are the effects of varying **a** the number of initial infections $I_W(0)$, **b** mean exposed times τ_E (days), **c** mean infectious times τ_I (days), and **d** different ratio of weak-symptom cases p_w. Each panel shows the dependence of the epidemic transition upon varying one of the model parameters and the original choices correspond to the black markers.
