# Supplementary material for: Discontinuous epidemic transition due to limited testing
Source: Nat Commun. 2021 May 10;12:2586. doi: 10.1038/s41467-021-22725-9 (PMC8110767; doi:10.1038/s41467-021-22725-9)
Supplement: Supplementary file 3 — Description of Additional Supplementary Files [file 41467_2021_22725_MOESM3_ESM.pdf]

Description of Additional Supplementary Files

File Name: Supplementary Video 1.

Description: Discontinuous flattening. **a** Daily new cases for continuously decreasing values of R0 (1.3 < R0 < 3) with active testing and quarantining. After an initial continuous flattening the epidemic curve suddenly drops to very small numbers of new cases. Testing capacity is limited to NT = 1000 tested individuals per day. **b** Daily new cases for decreasing values of R0 (0.85 < R0 < 1.5) without testing and quarantining. Decreasing progressively R0 gradually flattens the epidemic curve until a very low number of cases is reached. In both cases the population is P=3162×3162 and epidemics start with 100 initial weak-symptom infectious.
